# Supplementary figures and images for: Distribution and natural infection status of synantrophic triatomines (Hemiptera: Reduviidae), vectors of Trypanosoma cruzi, reveals new epidemiological scenarios for chagas disease in the Highlands of Colombia
Source: PLoS Negl Trop Dis. 2021 Jul 19;15(7):e0009574. doi: 10.1371/journal.pntd.0009574 (PMC8321397; doi:10.1371/journal.pntd.0009574)

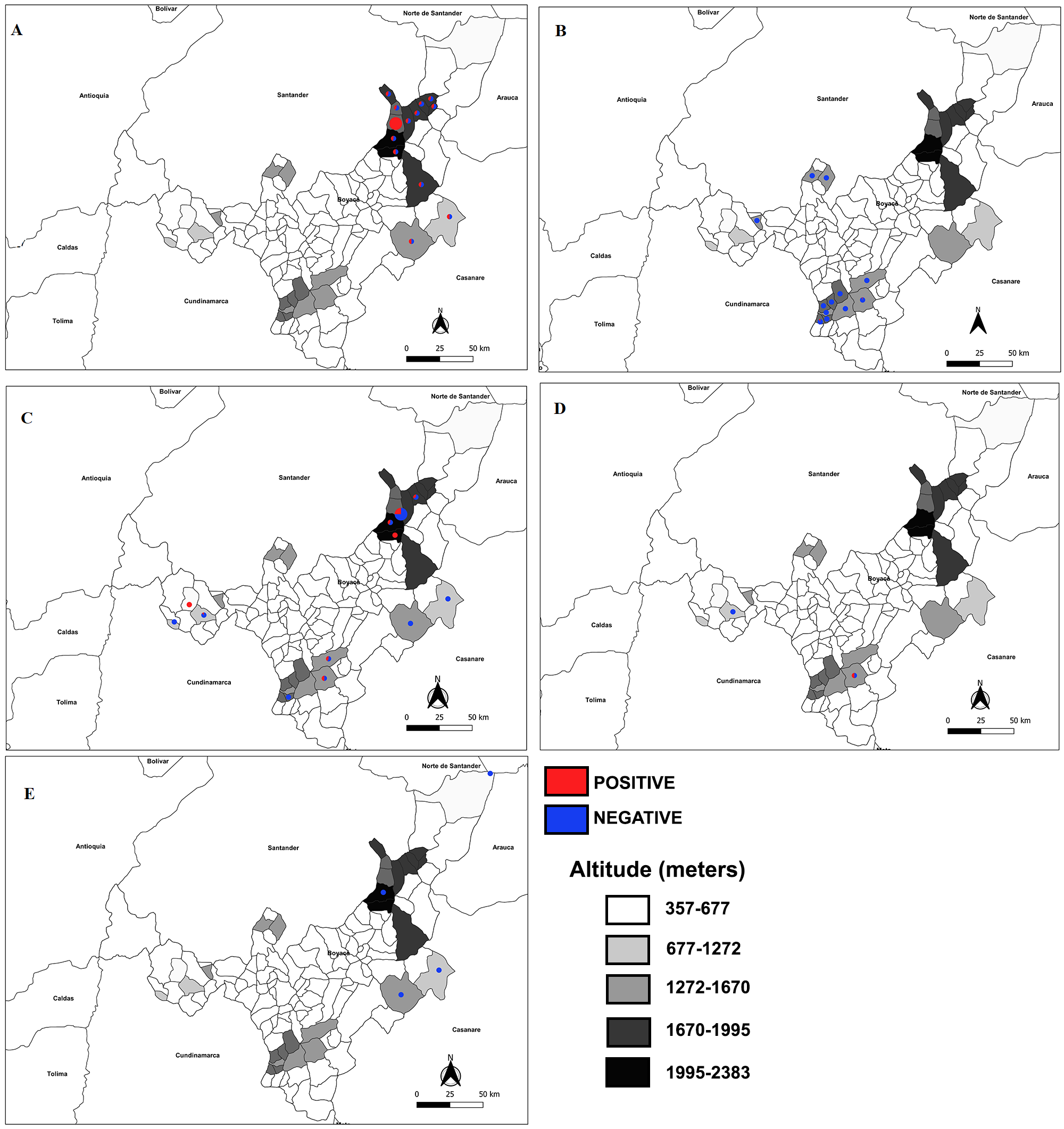

Supplement: S1 Fig — T. dimidiata (A), T. venosa (B), P. geniculatus (C), P. rufotuberculatus (D) and R. prolixus (E) from March 2017 to November 2018. The map was built using the free and open source QGIS software version 3.4 (https://www.qgis.org/en/site/forusers/download.html) and shapefiles were obtained from the free and open source DIVA-GIS site (https://www.diva-gis.org/gdata). (TIF) [file pntd.0009574.s001.tif]

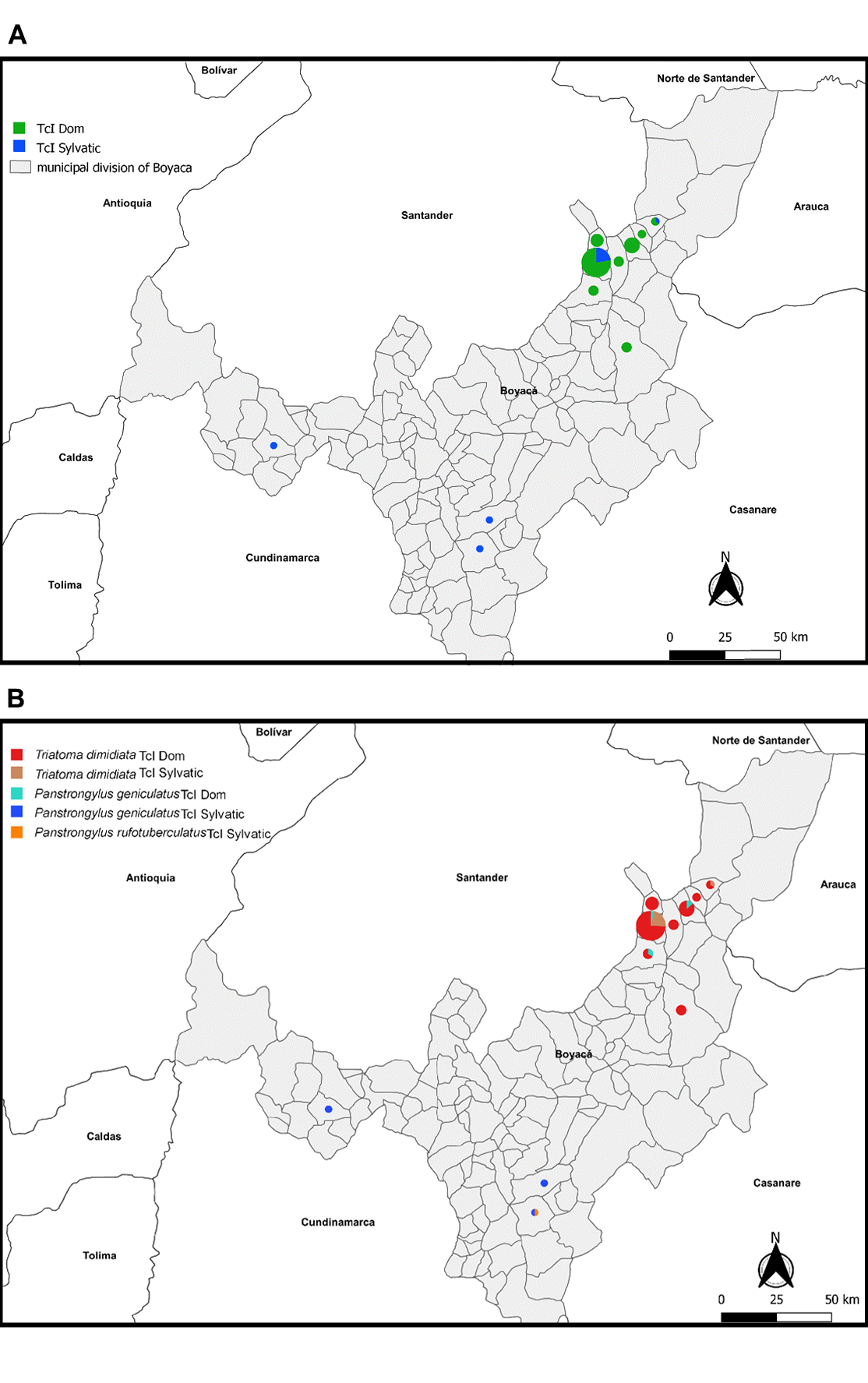

Supplement: S2 Fig — (A) TcIDom and TcI sylvatic. (B) TcIDom and TcI sylvatic by triatomines species. The map was built using the free and open source QGIS software version 3.4 (https://www.qgis.org/en/site/forusers/download.html) and shapefiles were obtained from the free and open source DIVA-GIS site (https://www.diva-gis.org/gdata). (TIF) [file pntd.0009574.s002.tif]
