# Supplementary material for: Distribution and natural infection status of synantrophic triatomines (Hemiptera: Reduviidae), vectors of Trypanosoma cruzi, reveals new epidemiological scenarios for chagas disease in the Highlands of Colombia
Source: PLoS Negl Trop Dis. 2021 Jul 19;15(7):e0009574. doi: 10.1371/journal.pntd.0009574 (PMC8321397; doi:10.1371/journal.pntd.0009574)
Supplement: Attachment — Submitted filename: Review.docx [file pntd.0009574.s003.docx]

The data presented in this article deserve to be published because they are the result of a lot of fieldwork and shed light on the current situation of the vector infestation in one of the most endemic regions for Chagas disease in Colombia. This study clearly shows that the risk of transmission of Chagas disease persists in the studied region. Also, the discussion and the conclusions of the authors, clearly show the need to examine and take into account the new transmission scenarios following the control program of domestic *R. prolixus*, which is until now, the main vector implicated in the transmission of the disease. However, this article needs improvement and I attach a series of minor and major remarks and corrections in the order of the text. In addition, it is essential to have the text revised by an English-speaking person.

*The line numbering we use in the text below corresponds to the text downloaded in .docx format from the pdf*

**Abstract**

*Find the following corrections in red and one queries in blue:*

**“Introduction:** The actualization of distribution and natural infection of triatomine bugs is key for planning, prioritize, and executing strategies for controlling Chagas disease (CD), especially after interruption of transmission by primary vectors. Boyaca, **is** becoming the department with the largest number of municipalities certified by PAHO free of intra-domestic transmission by *R. prolixus* in Colombia*.* **Also, the** present work aimed to determine the distribution, natural infection, and molecular characterization of *T. cruzi* in synanthropic triatomines from different municipalities in **this** department. **Materials and Methods:** Between 2017-2018, 29 municipalities from Boyaca were analyzed of which 72% (18/25) has been certified without *R. prolixus*, and 31% (9/29) are located above 2000 m. Triatomine bugs were collected through **two methods:** (*i*) active search carried out by technical personnel from the **health department**, and (*ii*) community participation through Triatomine **Collection** Stations (PITs-acronym in Spanish). For evaluation of natural infection, triatomines were morphologically identified and grouped in pools from one to five individuals of the same species and collected in the same household. DNA derived from feces of **these** pools were analyzed by PCR **for *T. cruzi* infection** using primers flanking the satellite DNA of the parasite, and SL-IR **primers** for differentiation of **TcI** of the others DTUS, and **the** identification **of genotypes**. **Results:** A total of 670 triatomine bugs belonging to five species: *Triatoma dimidiata* (73.2%), *T. venosa* (16.7%), *Panstrongylus geniculatus* (5.7%), *Rhodnius prolixus* (4.4%), and *P. rufotuberculatus* (0.4%), were collected in **the** 29 municipalities. **A total of 71.6% bugs were collected in intradomicile and the rest in peridomicile.** *T. dimidiata* was the most distributed **species with** the higher infection index (please add the infection index here), followed by *T. venosa* and *P. geniculatus*. TcI was the only DTU found, **with the TcIDom genotype** in 80% of positive samples and TcI sylvatic in **the others**. **Conclusions:** After **the** certified elimination of *R. prolixus* in some municipalities **in** the Boyaca department, *T. dimidiata* represents the greatest risk for CD transmission. However, *T. venosa*, *P. geniculatus,* and *P. rufotuberculatus* also **provide** the risk of transmission. Finally, the presence of residual *R. prolixus,* put at risk the success achieved. Newer transmission scenarios demand the application of alternative strategies that not only consider the elimination through of spraying house, but also, directed toward reducing triatomines entry into the houses, and active surveillance with community participation.”

**Author summary**

Line 38: change “*R. prolixus,* Colombia’s main vector” by “*R. prolixus,* the Colombia’s main vector”

Line 66: change “masl” by “m.a.s.l.” and also throughout the text.

**Introduction**

Line 58-59: change “and has been divided into six Discrete Typing Units (DTU's I-VI)” by “and has been divided into six discrete typing units (DTUs, TcI to TcVI)…

Line 65: change “the genera *Triatoma, Rhodnius* and *Panstrongylus* are the main vectors” by “the genera *Triatoma, Rhodnius* and *Panstrongylus* include the main vectors”

Line 67-68: change “due to their infestation indexes and vector capacity [8]” by “due to its infestation index and vector capacity [8]”

Line 80-81: change “However, the geographical position and orography of this department, configured by different biogeographic zones, determine the presence” by “However, the geographical position and orography of this department grant it different biogeographic zones which determine the presence…”

Line 84: I thing that you can’t use the abbreviations for *P. rufotuberculatus,* and *R. pictipes* because these species are cited for the first time in the text even if other specie of the same genus were cited before.

**Results**

**Major comments:**

1- In the “3.2 Infestation chapter” (i) I find it necessary to introduce the colonization index. It is a classic index which gives an idea of the capacity of a species to settle in a house and thus to probably become even more dangerous than if this species is only of incursion (ii) I also think that the presentation of the distribution of species according to ecotopes is missing here, the results of which appear in Table 3.
2- In the chapter 3.3 Natural infection and ecotopes It would be necessary to add a statistic to see if significant difference of triatomine infection exist between the two ecotopes, and do again the sentence according to the result.
3- In M & M you have a chapter “Detection of PCR inhibition” but no result are presented about this test. You need to give some results, with 1 or 2 sentences or delete this information.

**Minor corrections:**

Lines 96-98: Abbreviate the genera of the species.

Line 98: change “were collected at 29 municipalities” by “were collected in 29 municipalities”

Lines 101-102: This last sentence should be moved to the beginning of the paragraph: “Finally, 23.1% (155/670) of triatomines were collected in 2017 and 76.9% (515/670) in 2018.”

Line 103: change “*T. dimidiata* and *T. venosa* with a presence in” by “*T. dimidiata* and *T. venosa* which were present in”

Line 106: Remove Table 1 from the parenthesis because Table 1 presents the total results, but not the detail by species.

Lines 106-107: change “without *R. prolixus* (Table 1). The 58% (285/491) of collected triatomines of this specie were adults and the rest were nymphs.” By “without *R. prolixus* (Table 2), and 58% (285/491) of the collected triatomines were adults.

Lines 138: change “(Table 1).”by “(Table 1 and 2).”, because the information of the municipalities about species is in Table 2 and the information of altitudes of the municipalities is in Table 1.

Line 108: change “The later was found mainly of southwest provinces” by “The later was found mainly in southwest provinces”

Line 110: change “(Table 1). The 83% (93/112) “ by “(Table 2),.and 83% (93/112)”

Line 125: change “The entomological indices for the population were infestation index” by “The entomological indices overall municipalities were infestation index”

Line 128: add “municipalities” after “and Panqueba (4%)”

Line 128: You introduce here the result of a statistic but, (i) it is necessary to put the degrees of freedom (df), (ii) it is necessary to say what it is ... It seems that this statistic allows to say that the infestation rates of the municipalities cited are not homogeneous, you have to say it. This also apply line 201, please do a revision.

Line 141: change “Figures S1” by “Figure S1”

Lines 143 to 151: correction in red

Only TcI was found in positives samples, and both genotypes (TcIDom and TcI sylvatic) were detected (Figures 3A and 3B)…Whit respect to species, for *T. dimidiata* the 85% of the sequence corresponded to TcIDom, and the rest to TcI sylvatic, concerning *P. geniculatus, ,* equal number of samples were TcIDom and TcI sylvatic (50% (3/6) each), and the sequence derived from *P. rufotuberculatus* belong to the TcI sylvatic. …We did not find a mixture of both genotypes.

**Discussion**

**Major corrections:**

Lines 194 to 197. The last part of the sentence is not clear, we do not understand which are the two theories in balance. Moreover, the term "theories" is not appropriate, it is rather a hypothesis. You have to rewrite this sentence.

“… the results here found could be a consequence of *T. venosa* mobility since peridomicile area or consequence of a peculiar domiciliation of this triatomine species in this department [9] [27]. Therefore, we suggested that both theories should be explored.”

Minor corrections

Lines 155 and 186: change “inside home dwellings” by intradomicile; do the following correction: the presence of infected secondary vectors in intradomicile and peridomicile is the new scenario that…

Lines 169-171: delete (.) before (ii), (iii) and (iv). Delete Figure 2 because there is not information of species and our sentence relate to species. Delete finally it is not necessary.

Line 188: add “it” in the following part “where it has been related to”

Line 198: change “year” by “years”

Line 205: change “these” by “them”

**Materials and Methods**

**Major corrections**

1 - 4.2	Triatomine collection and processing

I have several queries for this part. The selection of the 29 municipalities is not clear. You mentioned in the previous chapter that the 29 municipalities under study had an “history of infestation by triatomines in the last 5 years”. In this part you specify that there is two groups of municipalities. The first one is a group of municipalities with certification done on 2019, but your study is during 2017-2018. What is the status of these municipalities at the time of the study? How are they selected in the department among all the municipalities? Moreover you must specify clearly what is the certification: free of transmission? free of domestic vectors? free of *R. prolixus*?. In the summary you indicate 18 municipalities have been certified free of *R. prolixus*, do they match the one with an asterisk in Table 1?

For the second group you do not indicate its status of infestation at the moment of the study and how they were selected among all the municipalities of the department. According to the following information “One entomological sampling per year was carried out in the municipalities of the first group, in the urban and rural areas of each municipality.” two collections were done in the first group (2017 and 2018) is it true? not clear in the text. For the second group you say that the triatomines collected by the residents were keep at the PITS. Are the inhabitants required to bring the triatomines to the PITS or does a member of the PITS visit houses with a certain frequency to collect triatomines? Could you clarify that.

2- Regarding DNA extraction, you report that the faeces are obtained by abdominal pressure, what have you done for the insects which arrived dead in the laboratory? what was their proportion? Have you done dissection? or do you have no deaths?

**Minor corrections**

Line 261: change “Andeans Mountains:” by “Andean Mountains:”

Lin265-267: see the following corrections in red

The topography of this area, shaped by the eastern mountains, determines the presence of diverse landscapes and climatic conditions, highlands (paramos) to hot climate areas (located less than 500 m.a.s.l.). This department…

Line 270: change “29 municipalities belong to nine provinces” by “29 municipalities belonging to nine provinces”

Line 271: change “following” by “according to”

Lines 283-285: see the following corrections in red

In brief, synanthropic triatomines were searched indoors and outdoors for 30 min (in each place?); flashlight was used to help seeing triatomines into cracks and crevices of building walls, behind pictures.

Line 287: “For the second group, insects were collected indoors (only?)

Line 299: change “By each pool,” by “For each pool,”

Line 300: add “,” after pH 7.2

Line 303, 304 and 311: *T. cruzi* must be italized… there is several other *T. cruzi* that are not italized in the text below, please revise

Line 314: change “discrimination of *T. cruzi* DTUI (TcI) of the other DTUs” by “discrimination of TcI DTU of the other DTUs”

Lines 323-325: change “Samples were considered as TcI when a band of 350 bp was observed in the gel.

For TcI genotype identification,” by

“TcI was identified in the samples when a band of 350 bp was observed in the gel.

For TcI positive samples,”

Line 348: change “These base maps were “ by “The base maps were”

Lines 351-352: the verb is missing in the sentence

Lines 352-356. This part must be move in the legend of the figures: “For this, relative … collected and examined species.

**Tables**

**Table 1:**

The title is not precise enough. Title proposition: Geographical data and entomological indexes of the 29 studied municipalities in Boyaca department (Colombia), from March 2017 to November 2018.

change “Number of infected houses” by “Number of infested houses”

Indicate at the foot of the table what means the asterisk after some municipalities.

**Table 2 :**

The title is not satisfactory. Title proposition: *T. cruzi* infection rates evaluated in triatomines collected in the 29 studied municipalities in Boyaca department (Colombia), from March 2017 to November 2018.

It would be easier for the reader if the order of the municipalities is the same as in Table 1

Indicate at the foot of the table what means the asterisk after some municipalities.

Indicate at the foot of the table what means “Pools analyzed”.

For the "total" line, some pourcentages are missing.

**Table 3**

The title is not satisfactory. Title proposition: *T. cruzi* infection rates evaluated in the triatomine species collected in intra- or peridomiciles in the 29 studied municipalities (Boyaca department, Colombia), from March 2017 to November 2018.

Change “Intradomicilie” by “Intradomicile”

Change “Peridomicilie” by “Peridomicile”

Change Not date by “Unknown origin” or “Intra- or peridomicile”

The genus of the species must be abreviated as in Table 2.

Indicate at the foot of the table what means “N”.

Indicate at the foot of the table what means “Pools analyzed”.

**Figure 1**

Change the beginning of the title : Distributions of triatomines collected in the 29 studied municipalities in Boyaca Department (Colombia) between 2017 and 2018.

It must be specified what the box represents

It must be indicate in the legend what the size of the circles corresponds (do it too in Figure 3).

I recommend that the size of department names be (to be applied in Figures 2 and 3) as well as that of municipality numbers.

Italized the names of species in the figure, do it too in Figure 3B

**Figure 2.**

Change the title as in Figure 1

As some circles overlap two municipalities, I suggest adding the numbers of the 29 municipalities studied in the figure.

**Figure 3.**

The title is not satisfactory. Title proposition: Geographical distribution of TcI DTU genotypes (TcIDom and TcI sylvatic), in the 29 studied municipalities in Boyaca department (Colombia) between 2017 and 2018. A. In municipalities . B In triatomine species. DTU = discrete typing unit.

**Figure S1**. Modify title according to the proposed modifications in the other figures.
