# Supplementary material for: Distribution and natural infection status of synantrophic triatomines (Hemiptera: Reduviidae), vectors of Trypanosoma cruzi, reveals new epidemiological scenarios for chagas disease in the Highlands of Colombia
Source: PLoS Negl Trop Dis. 2021 Jul 19;15(7):e0009574. doi: 10.1371/journal.pntd.0009574 (PMC8321397; doi:10.1371/journal.pntd.0009574)
Supplement: Attachment — Submitted filename: Reviewers_answerPLOSTND.docx [file pntd.0009574.s004.docx]

13 December, 2020

Dr.

Editor, PLOS NTDs.

RE: Review of the article PNTD-D-20-01337: "Distribution and Natural Infection status of Synantrophic Triatomines (Hemiptera: Reduviidae) Vectors of *Trypanosoma cruzi,* Reveals New Epidemiological Scenarios for Chagas Disease in Highlands of Colombia."

First, the authors wish to thank the reviewer and editor for their clear and concise comments. It is essential to state that most of the corrections were conducted according to reviewer recommendations.

We sincerely have adequately addressed all matters brought up by them, and we are much grateful to them for their time and attention.

Please find below the answers to the reviewers' evaluations.

Best Regards,

Dr. Omar Cantillo-Barraza

Corresponding author

*************************************************************************

Comments from the editors and reviewers:

Title: Distribution and Natural Infection status of Synantrophic Triatomines (Hemiptera: Reduviidae) Vectors of *Trypanosoma cruzi,* Reveals New Epidemiological Scenarios for Chagas Disease in Highlands of Colombia

**Dear Dr. Cantillo Barraza,**

Thank you very much for submitting your manuscript "Distribution and Natural infection of Synantrophic Triatomines (Hemiptera: Reduviidae) vectors of Trypanosoma cruzi reveals new epidemiological scenarios for Chagas disease in highlands of Colombia" for consideration at PLOS Neglected Tropical Diseases. As with all papers reviewed by the journal, your manuscript was reviewed by members of the editorial board and by several independent reviewers. In light of the reviews (below this email), we would like to invite the resubmission of a significantly-revised version that takes into account the reviewers' comments.

When resubmitting your manuscript, please carefully consider all issues mentioned in the reviewers' comments, outline every change made point by point, and provide suitable rebuttals for any comments not addressed.

**Methods**

Reviewer #1: The submitted article has a descriptive approach. The aim of the study is clear though the objectives could be refined for sake of clarity. Overall, the study design is appropriate to address the stated objectives. However, it is necessary to clarify the study population, give more details on the study design and some of the estimated indices. Additionally, I would suggest including a new PCR assay to differentiate all DTUs. Authors could better exploit the impressive amount of data collected with further spatial analysis. See my general comments.

Authors report having an approved protocol to analyze animal species.

Answer: The authors wish to thank the reviewer for (his/her) clear and concise comments. Below, we exposed our detailed responses.

*************************************************************************

**Results**

Reviewer #1: The results presented are in accordance with the planned analyzes.

See my general comments.

Answer: The authors wish to thank the reviewer for (his/her) clear and concise comments. Below, we exposed our detailed responses.

*************************************************************************

**Conclusions**

Reviewer #1: The discussion and conclusion could be improved by making a comparison of this problem with other countries, regions and vector species. The authors did not mention any limitations of the study. See my general comments.

Answer: The discussion and conclusions were strengthened, and the limitations of the study were highlighted.

*************************************************************************

**Editorial and Data Presentation Modifications?**

Reviewer #1: Please correct some typos, also please use italics for specific name and the correct word is "species" not "specie".

Answer: The authors made the correction suggested by the reviewer.

Please review the use of decimals throughout the work.

Answer: The authors made the correction suggested by the reviewer.

Please provide the necessary information in the figures to avoid having to go back to M&M.

Answer: The authors made the correction suggested by the reviewer.

Table 1. I think there is a mistake in the name of the column "Number of infected houses." Please explain as footnotes what

"-" means, the same to "Number household" column and "*" in municipality.

*Answer*: The Reviewer's recommendation was accepted. View table 1 and legends of table 1.

*************************************************************************

**Summary and General Comments**

Reviewer #1: The authors of the manuscript entitled "Distribution and Natural infection of Synantrophic Triatomines (Hemiptera: Reduviidae) vectors of Trypanosoma cruzi reveals new epidemiological scenarios for Chagas disease in highlands of Colombia" propose to describe the distribution of secondary vectors of Chagas disease after the quasi-elimination of the main vector in the Department of Boyaca, Colombia and thus assess the risk of vector transmission by them. The authors obtain infestation data and bugs from approximately 14,000 houses, using two different collection methods. In addition, authors evaluate the infection status, the frequency of T. cruzi genotypes, and map all this information at the municipality level. This topic was also described in seven other departments of Colombia by Hernandez et al. (2016) and I think it could be suggested as a widespread pattern in this country. I believe that the collected data and in part the proposed analysis have strong potential from the point of view of public health for the country and Chagas disease research in general. However, there are a number of issues that need to be addressed or clarified before publication.

*Answer*: We agree with the comment made by the evaluator, the distribution of infected vectors is wide and a general problem in Colombia.

Introduction:

- The authors could present a general aspect of the topic including bibliography on other countries and vector species. Also, include reference of the altitudinal distribution of the studied species.

*Answer*: The Reviewer's recommendation was accepted. In lines 76 to 86

M&M:

- Additional information on study area should be included. How were municipalities selected?

*Answer*: The Reviewer's recommendation was accepted. In issue 4.2, Triatomine collection and processing, lines: 320 to 344.

- There is not information on infestation by these vector species for the area before this study.

*Answer*: The scientific literature's information does not show indices of infestation or natural infection (Guhl et al. 2007, Parra et al. 2015). Additionally, in Boyaca Health, there are no previous records of the infestation rates of all the municipalities studied.

- It is not clear how many municipalities were certified by PAHO and the reference provided it does not seem to match.

*Answer*: The Reviewer's recommendation was accepted in issue 4.2. Triatomine collection and processing lines 320to 324. Twenty-four Boyaca municipalities have been certified without intra-domestic *R. prolixus* by PAHO in three different moments: 6 in 2010, 5 in 2017, and 13 in 2019.

- The study design should be clarified. The 29 municipalities were divided into two groups according to the vector capture method, but it is not clear which municipalities were included in which group.

*Answer*: The Reviewer's recommendation was accepted in issue 4.2, Triatomine collection and processing lines 320 to 336.

- It is necessary to clarify the entomological evaluation carried out for the second group and if it was used to estimate the infestation index despite the fact that the capture method differs between groups.

*Answer*: For the second group (municipalities where health workers of BDHS carried out no active search), the infestation index was not carried out because the collection methodology did not allow the calculation. It was clarified in the M&M lines 388 to 389 .

- The authors estimate an infection index through the examination of a pool of triatomines collected in the same house. However, it is not clear how it was calculated. Justify why the authors decided to use this infection index and not an insect prevalence of infection. Please revise if the informed estimation is an index or a real rate as it is called in the discussion.

*Answer*: The infection index (number of infected pools/total number of analyzed pools) *100. We calculate the infection outcomes at the municipality and species level. It was clarified in the M&M lines 388 to 395. On the other hand, we did not carry out a randomized sampling. Additionally, we did not know previous data on the infestation. It was impossible to calculate a representative sample size that would estimate the prevalence of entomological infection.

- The authors mention that they use the TCC-TC1-TC2 primers as described in Burgos et al. 2007 to discriminate TcI from the rest of the UDTs instead of Souto et al. 1996. This proposed protocol does not allow to differentiate TcI from TcIII or TcIV, because no amplification product is expected for the latest UDTs. In order to differentiate all UDTs I suggest reviewing the publication by Marcet el al. 2006., or reviewing again Burgos et al. 2007.

*Answer*: We accepted the evaluator's recommendation and made new ones using the primers reported by Ramirez et al. 2010, and also, we used part (PCR directed to the SL-IR region only) of the algorithm implemented by Hernández et al. 2016. The results of these new PCRs evidenced only the circulation of TcI. On the other hand, we corrected the reference because the primers used in this work were reported by Souto et al. 1996. Lines 363 - 380.

- In my opinion, the authors could give more support to the results including new statistical analyzes to evaluate the potential aggregation or association of the distribution of insects, as well as of the infestation and infection index.

*Answer*: We accepted the evaluator's recommendation, and a new Geospatial analysis was made, sew in the apart *4.8 Geospatial analysis.* Lines 410 to 431

- Line 351-352 says "Mapping of the triatomine distribution in the urban area of the 29 municipalities from the study area". Were only urban houses mapped?

*Answer*: We accepted the evaluator's recommendation, and we corrected because the maps were calculated based on the urban and rural information of each municipality. Please see Line 403.

**Discussion:**

- The discussion could be improved by including a more general overview of the problem and including comparisons with other studies involving the same or other vector species, both within Colombia and at the regional / global level.

*Answer*: We accepted the evaluator's recommendation, and we rewrite part of the discussion, including the comparisons suggested by the evaluator. Please see lines 186 to 222.

- The finding of T. dimidiata and P. geniculatus in municipalities located above their altitudinal range of distribution is very interesting as the authors pointed out. However, I found that in Guhl et al. 2007 (Reference 9) both species were previously found in these municipalities and were also found infected with T. cruzi. Please clarify this point.

*Answer*: It is crucial the evaluator's observations about the previous report of Guhl et al. 2007 (Trypanosoma spp) and Parra et al., 2015 (T. cruzi), in some of the municipalities that included this study. However, the present study was developed with a methodology that demonstrated: (i) levels of infestation of synanthropic vectors. (ii) significant natural infection levels (iii) determined that the domestic cycle is the most important in municipalities located over 2,000 m. (iv) we employed a robust sample size. This allows us to suggest that there is a significant risk of transmission in these municipalities, and it is not an isolated event. This was writing in the word line 76 to 86 and 212 to 222 and 281 to 291.

How do the authors interpret the risk of transmission by secondary vectors suggested with the almost nil seroprevalence in humans under 18 years old in the study area? Does the seroprevalence study match spatially with the area of greatest infestation detected in this work?

*Answer*: The Reviewer's recommendation was accepted, and the authors and we adjust the advice on lines 297 to 305.

The authors should include some limitations that they consider for the study.

*Answer*: the limitation of this study were include in the draft. Please see lines 292 to 305.

There is a problem with the references cited. In some cases, it does not seem to support the text (eg, Line 241: Cura et al. for altitudinal distribution of vector species; Line 200, references number 28 and 29; Line 243 Reference 36), and it seems that there is an error in the references order. I suggest reviewing them exhaustively.

I suggest avoiding the writing of textually similar phrases from recent works published by the main author. For example the final sentence of the discussion and the abstract.

*Answer*: the limitation of this study were include, and all references were adjusted. On the other hand, the indicated phrases were modified. L288 – 301.

*************************************************************************

**Reviewer #2: Review: Plos Tropical Neglected Diseases**

**Manuscript**

**General Comments: The manuscript entitled "Distribution and Natural infection of Synantrophic Triatomines (Hemiptera: Reduviidae) vectors of Trypanosoma cruzi reveals new epidemiological scenarios for Chagas disease in highlands of Colombia" presents relevant information on several species vectors of Trypanosoma cruzi in the Boyaca department of Colombia. The subject of the manuscript is in accordance and in the scope of PTND. However, several corrections and a new revised version must be considered before its acceptance for publication.**

**Specific comments: Editorial corrections must be made throughout the manuscript.**

**English review is deeply recommended**

- *Answer*: The reviewer's recommendation was accepted. Therefore, the paper was corrected by an English professional editor.

**Scientific names must be written in full in the beginning of a sentence or paragraph.**

- *Answer*: The reviewer's recommendation was accepted. Therefore, these items were corrected according to the reviewer's recommendation.

**Please check the scientific names throughout the manuscript. For example, check the lines 198, 215.**

- *Answer*: The reviewer's recommendation was accepted. Therefore, these items were corrected according to the reviewer's recommendation (L236, 255).

**Results / Discussion: Why a huge difference in the numbers of triatomines collected: "Finally, 23.1% (155/670) of triatomines were collected in 2017 and 76.9% (515/670) in 2018." This issue must be clarified and properly discussed.**

- *Answer*: The differences in triatomine collections between 2017 and 2018 could be related to some administrative problems that did not allow the early incorporation of health workers of BDHS, affecting the number of operational personnel for the development of the collections and the activation PRT. These reasons were exposed in the discussion at lines 292 to 297.

**Line 107 specie should be corrected: species; not just in this specific line but, throughout the whole manuscript**

- *Answer*: The reviewer's recommendation was accepted. Therefore, this mistake was corrected in the whole manuscript.

**Line 111 was founding should be corrected**

- *Answer*: The reviewer's recommendation was accepted. Therefore, this mistake was corrected in the manuscript (L120).

**Discussion**

**It would be interesting giving a broader view on the diversity of the Colombian triatomine vectors, for instance, mentioning how many species are recorded in the country, and how many of them are sylvatic however, eventually invade domiciles**

- *Answer*: About it, we consider that these aspects were mentioned in the introduction section (L68-75). According to the reviewer's recommendation, some modifications were made in this section (L76 to 86).

**Line 154 needs a reference**

- *Answer*: reference [16] was cited in this line (L188).

**Review the sentence 170-180**

- *Answer*: The reviewer's recommendation was accepted. Therefore, the paper was corrected by an English professional editor

**Line 196 replace" theory "by "possibility"**

- *Answer*: The reviewer's recommendation was accepted. Therefore, this item was corrected according to the reviewer's recommendation (L235).

**Materials and Methods**

**Line 284- Replace the "niche" by "ecotopes"**

- *Answer*: The reviewer's recommendation was accepted. Therefore, this item was corrected according to the reviewer's recommendation (L329).

**References: need extensive review**

- *Answer*: All references were checked according to the reviewer recommendation.

**Figs: Written information in the figs must be edited for better quality of the image**

- *Answer*: The reviewer's recommendation was accepted. The figures were edited

**Legends: Tables 1-3 Should include 'Colombia" all legends should be auto explanatory**

- *Answer*: The reviewer recommendation was accepted

**Figures caption: should be revised**

- *Answer*: The reviewer's recommendation was accepted. Figure captions were revised

*****************************************************************************

**Reviewer #3: Review: Plos Tropical Neglected Diseases**

**The data presented in this article deserve to be published because they are the result of a lot of fieldwork and shed light on the current situation of the vector infestation in one of the most endemic regions for Chagas disease in Colombia. This study clearly shows that the risk of transmission of Chagas disease persists in the studied region. Also, the discussion and the conclusions of the authors, clearly show the need to examine and take into account the new transmission scenarios following the control program of domestic R. prolixus, which is until now, the main vector implicated in the transmission of the disease. However, this article needs improvement and I attach a series of minor and major remarks and corrections in the order of the text. In addition, it is essential**

**"Introduction:** The actualization of distribution and natural infection of triatomine bugs is key for planning, prioritize, and executing strategies for controlling Chagas disease (CD), especially after interruption of transmission by primary vectors. Boyaca **is** becoming the department with the largest number of municipalities certified by PAHO free of intra-domestic transmission by *R. prolixus* in Colombia*.* **The** present work also aimed to determine the distribution, natural infection, and molecular characterization of *T. cruzi* in synanthropic triatomines from different municipalities in **this** department. **Materials and Methods:** Between 2017-2018, 29 municipalities from Boyaca were analyzed, of which 72% (18/25) have been certified without *R. prolixus*, and 31% (9/29) are located above 2000 m. Triatomine bugs were collected through **two methods:** (*i*) active search carried out by technical personnel from the **health department**, and (*ii*) community participation through Triatomine **Collection** Stations (PITs-acronym in Spanish). For evaluating natural infection, triatomines were morphologically identified and grouped in pools from one to five individuals of the same species and collected in the same household. DNA derived from feces of **these** pools was analyzed by PCR **for *T. cruzi* infection** using primers flanking the parasite's satellite DNA and SL-IR **primers** to differentiate **TcI** from the others DTUS and **the** identification **of genotypes**. **Results:** A total of 670 triatomine bugs belonging to five species: *Triatoma dimidiata* (73.2%), *T. venosa* (16.7%), *Panstrongylus geniculatus* (5.7%), *Rhodnius prolixus* (4.4%), and *P. rufotuberculatus* (0.4%), were collected in **the** 29 municipalities. **A total of 71.6% of bugs were collected in intradomicile, and the rest in peridomicile.** *T. dimidiata* was the most distributed **species with** the higher infection index (please add the infection index here), followed by *T. venosa* and *P. geniculatus*. TcI was the only DTU found, **with the TcIDom genotype** in 80% of positive samples and TcI sylvatic in **the others**. **Conclusions:** After **the** certified elimination of *R. prolixus* in some municipalities **in** the Boyaca department, *T. dimidiata* represents the greatest CD transmission risk. However, *T. venosa*, *P. geniculatus,* and *P. rufotuberculatus* also **provide** the risk of transmission. Finally, the presence of residual *R. prolixus* put at risk the success achieved. Newer transmission scenarios demand the application of alternative strategies that not only consider the elimination through of spraying house, but also, directed toward reducing triatomines entry into the houses, and active surveillance with community participation."

- *Answer*: all items were accepted (L2-34).

**Author summary**

**Line 38: change "R. prolixus, Colombia's main vector" by "R. prolixus, the Colombia's main vector"**

- *Answer*: the recommendation was accepted (L40)

**Line 66: change "masl" by "m.a.s.l." and also throughout the text.**

- *Answer*: All items were corrected according to reviewer recommendations (L51).

**Introduction**

**Line 58-59: change "and has been divided into six Discrete Typing Units (DTU's I-VI)" by "and has been divided into six discrete typing units (DTUs, TcI to TcVI).**

- *Answer*: This paragraph was corrected according to the reviewer's recommendation (L60-62).

**Line 65: change "the genera Triatoma, Rhodnius and Panstrongylus are the main vectors" by "the genera Triatoma, Rhodnius and Panstrongylus include the main vectors"**

- *Answer*: This paragraph was corrected according to the reviewer's recommendation (L67).

**Line 67-68: change "due to their infestation indexes and vector capacity [8]" by "due to its infestation index and vector capacity [8]"**

- *Answer*: This paragraph was corrected according to the reviewer's recommendation (L69-70).

**Line 80-81: change "However, the geographical position and orography of this department, configured by different biogeographic zones, determine the presence" by "However, the geographical position and orography of this department grant it different biogeographic zones which determine the presence…"**

- *Answer*: This paragraph was eliminated in the new version

**Line 84: I thing that you can't use the abbreviations for P. rufotuberculatus, and R. pictipes because these species are cited for the first time in the text even if other specie of the same genus were cited before.**

- *Answer*: These items were corrected according to the reviewer's recommendation (L90-91).

**Results**

**Major comments:**

**1-	In the "3.2 Infestation chapter" (i) I find it necessary to introduce the colonization index. It is a classic index which gives an idea of the capacity of a species to settle in a house and thus to probably become even more dangerous than if this species is only of incursion (ii) I also think that the presentation of the distribution of species according to ecotopes is missing here, the results of which appear in Table 3.**

- *Answer*: This paragraph was corrected according to reviewer recommendation, lines 134-139, table 1.

**2-	In the chapter 3.3 Natural infection and ecotopes It would be necessary to add a statistic to see if significant difference of triatomine infection exist between the two ecotopes, and do again the sentence according to the result.**

- *Answer*: The statistical test was included according to reviewer recommendations (152to 162 and L387 to 4397).

**3-	In M & M you have a chapter "Detection of PCR inhibition" but no result are presented about this test. You need to give some results, with 1 or 2 sentences or delete this information.**

- *Answer*: these results were included according to the reviewer's recommendation (L161-162).

**Minor corrections:**

**Lines 96-98: Abbreviate the genera of the species.**

- *Answer*: the recommendation was accepted (L100108).
- **Line 98: change "were collected at 29 municipalities" by "were collected in 29 municipalities"**
- *Answer*: the recommendation was accepted. This part was rewritten (L96)

**Lines 101-102: This last sentence should be moved to the beginning of the paragraph: "Finally, 23.1% (155/670) of triatomines were collected in 2017 and 76.9% (515/670) in 2018."**

- *Answer*: the recommendation was accepted (L105-L106).

**Line 103: change “*T. dimidiata* and *T. venosa* with a presence in” by “*T. dimidiata* and *T. venosa*** **which were present in”**

- *Answer*: the recommendation was accepted (L109).

**Line 106: Remove Table 1 from the parenthesis because Table 1 presents the total results, but not the detail by species.**

- *Answer*: *Answer*: the recommendation was accepted (L111).

**Lines 106-107: change "without *R. prolixus* (Table 1). The 58% (285/491) of collected triatomines of this specie were adults and the rest were nymphs." By** **"without *R. prolixus* (Table 2), and 58% (285/491) of the collected triatomines were adults.**

- *Answer*: the recommendation was accepted (L113-L114).

**Lines 108: change "(Table 1)." by "(Table 1 and 2).", because the information of the municipalities about species is in Table 2 and the information of altitudes of the municipalities is in Table 1.**

- *Answer*: the recommendation was accepted (L114).

**Line 108: change "The later was found mainly of southwest provinces" by** **"The later was found mainly in southwest provinces"**

- *Answer*: the recommendation was accepted (L109-114).

**Line 110: change "(Table 1). The 83% (93/112) "by "(Table 2),.and 83% (93/112)"**

- *Answer*: the recommendation was accepted (L116).

**Line 125: change "The entomological indices for the population were infestation index" by** **"The entomological indices overall municipalities were infestation index"**

- *Answer*: the recommendation was accepted (L132).

**Line 128: add** **"municipalities" after "and Panqueba (4%)"**

- *Answer*: the recommendation was accepted (L135).

**Line 128:** **You introduce here the result of a statistic but, (i) it is necessary to put the degrees of freedom (df), (ii) it is necessary to say what it is ... It seems that this statistic allows to say that the** **infestation rates of the municipalities cited are not homogeneous, you have to say it. This also apply line 201, please do a revision.**

- *Answer*: the reviewer recommendations were accepted (L136 -150).

**Line 141: change "Figures S1" by "Figure S1."**

- *Answer*: the recommendation was accepted (L161).

**Lines 143 to 151: correction in red**

**Only TcI was found in positives samples, and both genotypes (TcIDom and TcI sylvatic) were detected (Figures 3A and 3B)…Whit respect to species,** **for *T. dimidiata* the 85% of the sequence corresponded to TcIDom, and the rest to TcI sylvatic****, concerning *P. geniculatus,*equal number of samples were TcIDom and** **TcI sylvatic (50% (3/6) each), and the sequence derived from *P. rufotuberculatus* belong to the TcI sylvatic. …****We did not find a mixture of both genotypes.**

- *Answer*: the reviewer recommendations were accepted (L163-171).

**Discussion**

**Major corrections:**

**Lines 194 to 197. The last part of the sentence is not clear, we do not understand which are the two theories in balance. Moreover, the term "theories" is not appropriate, it is rather a hypothesis. You have to rewrite this sentence.**

**"… the results here found could be a consequence of *T. venosa* mobility since peridomicile area or consequence of a peculiar domiciliation of this triatomine species in this department [9] [27]. Therefore, we suggested that both theories should be explored."**

- *Answer*: The reviewer's recommendation was accepted. Therefore, this item was corrected according to the reviewer recommendation (L235).

**Minor corrections**

**Lines 155 and 186: change "inside home dwellings" by** **intradomicile; do the following correction****: the presence of infected secondary vectors in intradomicile and peridomicile is the new scenario that…**

- *Answer*: the reviewer recommendations were not accepted, because this word was removed (L186 to 188)

**Lines 169-171: delete (.) before (ii), (iii) and (iv). Delete Figure 2 because there is not information of species and our sentence relate to species. Delete finally it is not necessary.**

- *Answer*: This paragraph was eliminated in the new version

**Line 188: add "it" in the following part "where it has been related to"**

- *Answer*: the reviewer recommendations were accepted (L227).

**Line 198: change "year" by "years"**

- *Answer*: This paragraph was eliminated in the new version

**Line 205: change "these" by** **"them"**

- *Answer*: the reviewer recommendations were accepted (L246).

**Materials and Methods**

**Major corrections**

**1 - 4.2	Triatomine collection and processing**

**I have several queries for this part. The selection of the 29 municipalities is not clear. You mentioned in the previous chapter that the 29 municipalities under study had an "history of infestation by triatomines in the last 5 years". In this part you specify that there is two groups of municipalities. The first one is a group of municipalities with certification done on 2019, but your study is during 2017-2018. What is the status of these municipalities at the time of the study? How are they selected in the department among all the municipalities? Moreover you must specify clearly what is the certification: free of transmission? free of domestic vectors? free of R. prolixus?. In the summary you indicate 18 municipalities have been certified free of R. prolixus, do they match the one with an asterisk in Table 1?**

**For the second group you do not indicate its status of infestation at the moment of the study and how they were selected among all the municipalities of the department. According to the following information "One entomological sampling per year was carried out in the municipalities of the first group, in the urban and rural areas of each municipality." two collections were done in the first group (2017 and 2018) is it true? not clear in the text. For the second group you say that the triatomines collected by the residents were keep at the PITS. Are the inhabitants required to bring the triatomines to the PITS or does a member of the PITS visit houses with a certain frequency to collect triatomines? Could you clarify that.**

- *Answer*: The Reviewer's recommendation was accepted. all clarifications were included in issue 4.2 Triatomine collection and processing lines 319 to 336.

**2- Regarding DNA extraction, you report that the faeces are obtained by abdominal pressure, what have you done for the insects which arrived dead in the laboratory? what was their proportion? Have you done dissection? or do you have no deaths?**

*Answer*: the reviewer recommendations were accepted. We had a mistake in the redaction because all triatomines evaluated in this study arrived dead in our laboratory. This was clarified in lines 346 to 351.

**Minor corrections**

**Line 261: change "Andeans Mountains:" by** **"Andean Mountains:"**

- *Answer*: This paragraph was eliminated in the new version

**Lin265-267: see the following corrections in red**

**The topography of this area, shaped by the eastern mountains, determines the presence of diverse landscapes and climatic conditions, highlands (paramos) to hot climate areas (located less than 500 m.a.s.l.). This department…**

- *Answer*: the reviewer recommendations were accepted (L308-312).

**Line 270: change "29 municipalities belong to nine provinces" by** **"29 municipalities belonging to nine provinces"**

- *Answer*: the reviewer recommendations were accepted (L316).

**Line 271: change "following" by** **"according to"**

- *Answer*: the reviewer recommendations were accepted (L317).

**Lines 283-285: see the following corrections in red**

**In brief, synanthropic triatomines were searched indoors and outdoors for 30 min (in each place?); flashlight was used to help seeing triatomines into cracks and crevices of building walls, behind pictures.**

- *Answer*: the reviewer recommendations were accepted (L328 to 331)

**Line 287: "For the second group, insects were collected indoors (only?)**

*Answer*: the reviewer recommendations were accepted. For the second group, were collected indoors and peridomiciles (lines 338to 344)

**Line 299: change "By each pool," by** **"For each pool,"**

- *Answer*: the reviewer recommendations were accepted (L347).

**Line 300: add "," after pH 7.2**

- *Answer*: This paragraph was eliminated in the new version

**Line 303, 304 and 311: *T. cruzi* must be italized… there is several other *T. cruzi* that are not italized in the text below, please revise**

- *Answer*: the reviewer recommendations were accepted.

**Line 314: change "discrimination of *T. cruzi* DTUI (TcI) of the other DTUs" by** **"discrimination of TcI DTU of the other DTUs"**

- *Answer*: the reviewer recommendations were accepted (L373-374).

**Lines 323-325: change** **"Samples were considered as TcI when a band of 350 bp was observed in the gel.** **For TcI genotype identification," by** **"TcI was identified in the samples when a band of 350 bp was observed in the gel.** **For TcI positive samples,"**

- *Answer*: the reviewer recommendations were accepted (L384-383).

**Line 348: change "These base maps were "by** **"The base maps were"**

- *Answer*: the reviewer recommendations were accepted (L399).

**Lines 351-352: the verb is missing in the sentence**

- *Answer*: the reviewer recommendations were accepted (L4039).

**Lines 352-356. This part must be move in the legend of the figures: "For this, relative … collected and examined species.**

*Answer*: the reviewer recommendations were

**Tables**

**Table 1:**

The title is not precise enough. Title proposition: Geographical data and entomological indexes of the 29 studied municipalities in Boyaca department (Colombia), from March 2017 to November 2018.

change "Number of infected houses" by "Number of infested houses" ok

*Answer*: the reviewer recommendations were accepted

Indicate at the foot of the table what means the asterisk after some municipalities.

*Answer*: the reviewer recommendations were accepted, please see legends

**Table 2 :**

The title is not satisfactory. Title proposition: *T. cruzi* infection rates evaluated in triatomines collected in the 29 studied municipalities in Boyaca department (Colombia), from March 2017 to November 2018.

*Answer*: the reviewer recommendations were accepted, please see legends

It would be easier for the reader if the order of the municipalities is the same as in Table 1.

*Answer*: the reviewer recommendations was accepted, please see table 2

Indicate at the foot of the table what means the asterisk after some municipalities

*Answer*: the reviewer recommendations was accepted, please see legends

Indicate at the foot of the table what means "Pools analyzed". Ok

*Answer*: the reviewer recommendations were accepted; please see legends

For the "total" line, some pourcentages are missing. Ok

*Answer*: the reviewer recommendations were accepted, please see in table 2

**Table 3**

The title is not satisfactory. Title proposition: *T. cruzi* infection rates evaluated in the triatomine species collected in intra- or peridomiciles in the 29 studied municipalities (Boyaca department, Colombia), from March 2017 to November 2018.

*Answer*: the reviewer recommendations were accepted. Please see legends

Change “Intradomicilie” by “Intradomicile” ok

Change “Peridomicilie” by “Peridomicile” ok

Change Not date by "Unknown origin" or "Intra- or peridomicile" ok

The genus of the species must be abreviated as in Table 2. ok

Indicate at the foot of the table what means "N".

*Answer*: the reviewer recommendations were accepted, N was changed by NT (Number of Triatomines). Please see table 3 and legends

Indicate at the foot of the table what means "Pools analyzed". ok

**Figure 1**

Change the beginning of the title: Distributions of triatomines collected in the 29 studied municipalities in Boyaca Department (Colombia) between 2017 and 2018.

*Answer*: the reviewer recommendations were accepted. Please see the beginning of the title

It must be specified what the box represents

*Answer*: the reviewer recommendations were accepted. Please see the legends

It must be indicate in the legend what the size of the circles corresponds (do it too in Figure 3).

*Answer*: the reviewer recommendations were accepted. Please see the legends

I recommend that the size of department names be (to be applied in Figures 2 and 3) as well as that of municipality numbers.

*Answer*: the reviewer recommendations were accepted. Please see imagens

Italized the names of species in the figure, do it too in Figure 3B

*Answer*: the reviewer recommendations were accepted, all scientific names were Italicized

**Figure 2.**

Change the title as in Figure 1

As some circles overlap two municipalities, I suggest adding the numbers of the 29 municipalities studied in the figure.

*Answer*: the reviewer recommendations were accepted. Please see the legends of figure 2

**Figure 3.**

The title is not satisfactory. Title proposition: Geographical distribution of TcI DTU genotypes (TcIDom and TcI sylvatic), in the 29 studied municipalities in Boyaca department (Colombia) between 2017 and 2018. A. In municipalities. B In triatomine species. DTU = discrete typing unit.

*Answer*: the reviewer recommendations were accepted. Please see the legend of figure S2

**Figure S1**. Modify title according to the proposed modifications in the other figures.

*Answer*: the reviewer recommendations were accepted. Please see the legend of figure S2

**Sincerely,**

**Dr. Omar Cantillo-Barraza.**
