# Supplementary material for: Distribution and natural infection status of synantrophic triatomines (Hemiptera: Reduviidae), vectors of Trypanosoma cruzi, reveals new epidemiological scenarios for chagas disease in the Highlands of Colombia
Source: PLoS Negl Trop Dis. 2021 Jul 19;15(7):e0009574. doi: 10.1371/journal.pntd.0009574 (PMC8321397; doi:10.1371/journal.pntd.0009574)
Supplement: Attachment — Submitted filename: Reviewers_answerPLOSTND.docx [file pntd.0009574.s005.docx]

12 May, 2021

Dr.

Editor, PLOS NTDs.

RE: Review of the article PNTD-D-20-01337: “Distribution and Natural Infection of Synantrophic Triatomines (Hemiptera: Reduviidae) Vectors of *Trypanosoma cruzi* Reveals New Epidemiological Scenarios for Chagas Disease in Highlands of Colombia”.

First, the authors wish to thank the reviewer and editor for their clear and concise comments. It is important to state that most of corrections were conducted according to reviewer recommendations.

We hope sincerely have adequately addressed all matters brought up by them, and we are much grateful to them for their time and attention.

Please find below the answers to the reviewers’ evaluations.

Best Regards,

Dr. Omar Cantillo-Barraza

Corresponding author

*************************************************************************

Comments from the editors and reviewers:

Title: Distribution and Natural infection of Synantrophic Triatomines (Hemiptera: Reduviidae) vectors of Trypanosoma cruzi reveals new epidemiological scenarios for Chagas disease in highlands of Colombia

**Dear Dr. Cantillo Barraza,**

Thank you very much for submitting your manuscript "Distribution and Natural Infection Status of Synantrophic Triatomines (Hemiptera: Reduviidae), Vectors of Trypanosoma cruzi, Reveals New Epidemiological Scenarios for Chagas Disease in the Highlands of Colombia" for consideration at PLOS Neglected Tropical Diseases. As with all papers reviewed by the journal, your manuscript was reviewed by members of the editorial board and by several independent reviewers. The reviewers appreciated the attention to an important topic. Based on the reviews, we are likely to accept this manuscript for publication, providing that you modify the manuscript according to the review recommendations.

*************************************************************************

**Summary and General Comments**

Reviewer #1: The authors made strides in the readability of the manuscript (ms) and overall ms has improved a lot. However, there are still several important corrections and some issues that need to be clarified before the ms can be accepted for publication.

**Introduction**:

- I find somehow confusing how the certification of the municipalities from Boyacá department is named throughout the manuscript. Which is the correct epidemiological scenario: free of intra-domestic transmission; free of domestic R. prolixus; free of R. prolixus? Reference 24 refers to the certification of the interruption of the domiciliary transmission of Trypanosoma cruzi by Rhodnius prolixus.

*Answer*: The Reviewer´s recommendation was accepted. We made the homogenization the term according with epidemiological scenario (interruption of intradomiciliary T. cruzi transmission by R. prolixus) in the lines 6 - 7, 29-30, 40 - 41, 43 - 44, 73 -74, 89 - 90, 191 -192, 204-205, 281-282, 337 - 339.

- In the same way, please check if references 10 and 11 support the sentence on line 71-73. Is the statement “whose efforts have resulted in the certified elimination of domestic R. prolixus in 63 municipalities in 6 departments” correct?
- *Answer*: The Reviewer´s recommendation was accepted: line 73, and the reference was changed and the reference were revised

**Results:**

- The selection of municipalities remains a little confusing. In Mat&Met and at the beginning of the Results, the authors indicate that 52 municipalities were included in the study and were divided into two groups. However triatomines were found only in 29 municipalities. So, later in results the authors indicate that the municipalities studied are the 29 in which infestation was found. Is this correct? I would suggest including the municipalities from the first group in which no infestation was found for the calculations of the entomological indices.

*Answer*: The Reviewer´s recommendation was accepted. In the lines 101 to107 we described the results according to each group. In additions we include in the table 1, other six municipalities where active search was done but their did not infestation. Finally, the infestation index was recalculated (line 139).

- Line 106, please indicate what proportion of adult insects were found intradomicile.

*Answer*: The Reviewer´s recommendation was accepted. Lines 109 to 112.

- Line 146. I think the appropriate metric is "relative frequency" rather than "distribution", and it could be biased by the abundance of T. dimidiata.

*Answer*: The Reviewer´s recommendation was accepted. Lines 150.

- On the other hand, if the proportion of vectors of a given species found intradomicile was calculated, it would show that 100% of the R. prolixus were captured in this ecotope. Could this metric be more informative?

*Answer*: we consider that the information presented is relevant because for the municipality of Cubará, due to its geographical location the presence of *R. prolixus* on palms of the species Attalea has been reported by agents from the Boyaca Department Health Service (BDHS).

•	Line 161, Table 3 and Fig SI The statistical test employed to evaluate significant differences is missing.

*Answer*: The Reviewer´s recommendation was accepted, in the table 3 the statistical value was included.

Discussion

•	The ending of the first paragraph of the discussion should be improved. I do not understand how the diversity of triatomines will allow the identification of areas of action. Other named characteristics such as vector role in the transmission, species distribution, levels of infestation and infection could serve to stratify the areas according to risk of transmission.

*Answer*: The Reviewer´s recommendation was accepted, the ending of first was improved. Lines 199 to 201.

•	I suggest closing the manuscript with a paragraph of conclusions. For instance, the relevance of the herein presented results for the maintenance of transmission control in Colombia and other countries in similar situations could be included.

*Answer*: The Reviewer´s recommendation was accepted, a new paragraph was include. Lines 315 to 321.

•	Line 203, which are the previous levels of intra-domestic infestation by T. dimidiata in Boyaca or other places? Without this piece of information how can the authors support the hypothesize of “an advanced domiciliation process”? Could dwellers bias their catches towards this ecotope as has been recorded for T. infestans?

*Answer*: The Reviewer´s recommendation was accepted. We include entomological index of others authors for this department and Santander Departments. Lines 208-212.

•	Line 220-222. The results of this work, although very interesting, suggest the participation of these vectors in domestic transmission. In my opinion new studies are needed to assess the risk of infection in humans.

*Answer*: The Reviewer´s recommendation was accepted. Lines 226-228.

•	Line 265, the results presented "suggest" a high transmission risk.

*Answer*: The Reviewer´s recommendation was accepted. Lines 263-265.

•	Line 288-290, “We suggest expanding the altitudinal range to improve our understanding of the true entomological range of these vectors.” It has already been reported that these species have a higher altitudinal distribution than 2000 masl (in Colombia and other countries). May be, what merits further research is to describe the domestic transmission above 2000 masl in Colombia.

*Answer*: The Reviewer´s recommendation was accepted. Lines 289 to 297.

•	Line 299-303. What was the seroprevalence obtained by the 2019 BDHS study in children under 8 years of age?

*Answer*: Between 2017-2019 BDHS study 7,463 children under 15 years old and the seroprevalence found was of 4,01*10.000 children.

On the other hand, between 2008 to 2019 a total of 20.571 children under 15 years old were evaluated and 17 were infected for a general seroprevalence of 8,24 *10.000 children under 15 years old

M&M

•	I think it could be useful to clarify the definition of "intra-domestic" and "domestic"

*Answer*: The Reviewer´s recommendation was accepted, we correct and homogenized. The Intradomestic term was not use. While we use intradomiciliary to refer to the place where the insects are collected or the place where transmission could occur. Finally, the term domestic refers to the transmission cycle.

•	There is something wrong in the expression of the inclusion of both municipalities (i.e. Maripi and Cubara). On the other hand, Table 1 does not show infestation data for R. prolixus.

*Answer*: Cubara and Maripi municipalities were included in the group of active search for triatomines collection because they had recent reports of *R. prolixus* in the 2014 and 2015 according with entomological surveillance program of vector control program of the Boyaca Department Health Service (BDHS). Table 1.

**Minor comments**

•	Line 70 please replace with “natural infection rates”. Also, I think reference 9 is better for this sentence than reference 8.

*Answer*: The Reviewer´s recommendation was accepted. Lines 69.

•	Line 77, I think the authors want to say “below 2,000 m.a.s.l.”

*Answer*: The Reviewer´s recommendation was accepted. Lines 77

•	Line 101, “19 of these had been certified…(Table 1)” But in table 1 only 18 are marked with asterisk. Also, Maripi and Cubara are not marked with an asterisk.

*Answer*: The Reviewer´s recommendation was accepted. Table1. Maripi and Cubara are not marked with an asterisk because they were not certified free of *T. cruzi* intradomiciliary transmission by R. prolixus. However, they municipalities were evaluated through of Active search (table 1).

•	Line 105, Table 2 does not indicate the number of collected triamines. Line 106

*Answer*: The Reviewer´s recommendation was accepted.

•	Line 139, please eliminate the phrase “suggesting that infestation rates in the cities are not homogeneous”

*Answer*: The Reviewer´s recommendation was accepted.

•	Line 140, the sentence “The highest colonization indexes were found in Socota (100), Paya (100), Cubara (66.6), and Soata (50.1)” repeats the information given in lines 136-137.

•	Line 165, please replace for “Figure S2A and S2B”

*Answer*: The Reviewer´s recommendation was accepted.

•	Line 166, please check the text font.

*Answer*: The Reviewer´s recommendation was accepted. Lines 170 to 172

•	Line 187, the word "transmission" is repeated.

*Answer*: The Reviewer´s recommendation was accepted.

•	Line 189, consider using the term “distribution” rather than “concentration”

*Answer*: The Reviewer´s recommendation was accepted. Line 192

•	Line 190, please include “The risk of T. cruzi by secondary vectors”

*Answer*: The Reviewer´s recommendation was accepted. Line 196

•	Line 207, please rephrase as a conditional sentence.

*Answer*: The Reviewer´s recommendation was accepted. Line 214

•	Line 354, please check the appropiateness of reference 20 in this sentence. Also for reference 28 (Ramirez 2010 must be included).

*Answer*: The Reviewer´s recommendation was accepted. Lines 369

Reviewer #2: Plos NTD

Manuscript

General Comments:

The manuscript entitled: “ Distribution and Natural Infection Status of Synantrophic Triatomines (Hemiptera: Reduviidae), Vectors of Trypanosoma cruzi, Reveals New Epidemiological Scenarios for Chagas Disease in the Highlands of Colombia” has improved significantly. However, before its final acceptance a few corrections and changes must be carried out. I recommend a fine English and editorial review.

Introduction

- Number of triatomine species is not updated. Please correct 150 + (References)

*Answer*: The Reviewer´s recommendation was accepted. Line 66

- Line 70 – Correct “naturla” for “natural”

*Answer*: The Reviewer´s recommendation was accepted. Line 69

Results

- Line 110 – Cross out “Triatomines”

*Answer*: The Reviewer´s recommendation was accepted. Line115-116

Line 164 - Only TCI was found in positive samples. I would suggest cross out positive samples.

*Answer*: The Reviewer´s recommendation was accepted. Line 168

**Discussion**

- Line 186 - Data of the certification

Answer: The Reviewer´s recommendation was accepted. Line 190-192

- Line 287 – Change “ suggest “ by “ recommend “

*Answer*: The Reviewer´s recommendation was accepted. Line 295

- I am not sure that the authors carried the characterization of “spatial concentration” of secondary vectors since just sparce field captures were performed. I would say the authors recorded the presence of secondary vectors in 24 municipalities of Boyaca. Please recheck and use the most precise term for defining study carried out.

*Answer*: The Reviewer´s recommendation was accepted. Line 193

Line 202- Spaces are missing.

*Answer*: The Reviewer´s recommendation was accepted.

- Lines 256, 257- “ This species has the ability to develop peridomicile and domestic colonies in both the lowland areas and above 3,600 m.a.s.l. in Bolivia [22]. This sentence need several corrections: I would suggest changing it for: This species can be found in domiciliary and peridomiciliary ecotopes in the lowland areas and also above 3,600 m.a.s.l. in Bolivia [22].

*Answer*: The Reviewer´s recommendation was accepted. Lines 263 to 265

- It seems to me that the term “ability” is very anthropomorphic. Also in the original sentence the correct would be domiciliary and peridomiciliary colonies.

*Answer*: The Reviewer´s recommendation was accepted. Line 259

- The term domestic is not the best term at all. Please check the meaning of domestic. The colony could be inside the house but the domestication process could not be necessarily involved. Use whenever possible the term “domiciliary” instead of “domestic”.

*Answer*: The Reviewer´s recommendation was accepted. 6 - 7, 29-30, 40 - 41, 43 - 44, 73 -74, 89 - 90, 191 -192, 204-205, 281-282, 339 - 341.

- Lines 284-286 - Here, however, we found populations of T. dimidiata and P. geniculatus infected with T. cruzi in municipalities located higher than the recommended altitude. We would suggest using “previous recorded altitude.”

*Answer*: The Reviewer´s recommendation was accepted. Lines 289 to 291

Caveats- Are well presented and discussed. I just would include a relevant topic related to the frequency of the field captures to check for the species populations oscillations along the year and seasons.

*Answer*: The Reviewer´s recommendation was accepted. Lines 313 - 315

M & M-

- I could not find an explicit information related to the criteria used for the insect identification. Please include a reference

*Answer*: The Reviewer´s recommendation was accepted. Line 337-366

**Sincerely,**

**Dr. Omar Cantillo-Barraza.**
